# Electro-acupuncture reduced steatosis on MRI-PDFF in patients with non-alcoholic steatohepatitis: a randomized controlled pilot clinical trial

**DOI:** 10.1186/s13020-023-00724-w

**Published:** 2023-02-24

**Authors:** Jingjie Zhao, Qianyi Wang, Xinyu Zhao, Lina Wu, Juanjuan Li, Wen Zhang, Shuai Xu, Chaoru Han, Yi Du, Xiaofei Tong, Weijia Duan, Di Cao, Hao Ren, Xinyan Zhao, Xiaojuan Ou, Jidong Jia, Hong You

**Affiliations:** 1 Liver Research Center, Beijing Friendship Hospital, Capital Medical University, National Clinical Research Center of Digestive Diseases, No. 95 Yong-an Road, Xi-Cheng District, Beijing, 100050 China; 2 Clinical Center for Metabolic Associated Fatty Liver Disease, Capital Medical University, Beijing, China; 3 Department of Traditional Chinese Medicine, Beijing Friendship Hospital, Capital Medical University, Beijing, China; 4 Clinical Epidemiology and EBM Unit, Beijing Friendship Hospital, Capital Medical University, Beijing, China; 5 Department of Radiology, Beijing Friendship Hospital, Capital Medical University, Beijing, China

**Keywords:** Electro-acupuncture, Non-alcoholic steatohepatitis (NASH), Randomized controlled trial (RCT), MRI-PDFF

## Abstract

**Background:**

Non-alcoholic steatohepatitis (NASH) had not yet been approved therapy. Electro-acupuncture (EA) has been reported to have potential efficacy. However, high-quality clinical evidence was still lacking.

**Methods:**

NASH patients were randomized and allocated to either sham acupuncture (SA) or EA group in a 1:1 ratio, with the patient blinded. Each patient received 36 sessions of SA or EA treatment over 12 weeks, followed by additional 4 weeks. The primary outcome was the changes in relative liver fat content measured by magnetic resonance imaging proton density fat fraction (MRI-PDFF).

**Results:**

A total of 60 patients were enrolled. From baseline to week 12, the reduction of relative liver fat content measured by MRI-PDFF in the EA group (− 33.6%, quantile range: − 52.9%, − 22.7%) was significantly more significant than that in the SA group (− 15.8%, quantile range: − 36.1%, − 2.7%) (*p* = 0.022). Furthermore, the EA group had more patients who achieved MRI-PDFF to 30% reduction at week 12 (53.3% vs. 25.9%, *p* = 0.035). EA treatment also significantly reduced body weight (− 3.0 vs. + 0.1 kg, *p* = 0.034) and BMI (− 1.5 vs. − 0.2 kg/m^2^, *p* = 0.013) at week 16. Except for AST (− 27.4 vs. − 16.2 U/L, *p* = 0.015), other biochemical varieties, including ALT, fasting-glucose, cholesterol, and triglyceride, showed no statistically significant difference. Both groups measured no significant changes in liver stiffness by magnetic resonance elastography (MRE). There were no serious adverse events in either group.

**Conclusions:**

Twelve weeks of EA effectively and safely reduces relative liver fat content in NASH patients. Further multicenter randomized controlled studies are needed.

*Trial registration* Chinese Clinical Trial Registry, ChiCTR2100046617. Registered 23 May 2021, http://www.chictr.org.cn/edit.aspx?pid=127023&htm=4

**Supplementary Information:**

The online version contains supplementary material available at 10.1186/s13020-023-00724-w.

## Introduction

Non-alcoholic fatty liver disease (NAFLD) is one of the most frequent causes of chronic liver disease worldwide, with a prevalence of 29.8% [1]. In China, a dramatic increase in the overall prevalence of NAFLD and its proportion in liver disease was projected [2]. Non-alcoholic steatohepatitis (NASH) is the progressive form of NAFLD characterized by hepatic steatosis, inflammation, ballooning, and fibrosis. It occurs in up to 30% of NAFLD patients and carries an increased risk of cirrhosis, liver decompensation, and hepatocellular carcinoma (HCC) [3].

The lack of approved pharmacological interventions for NASH makes lifestyle modifications such as diet management and physical exercise the cornerstone for managing this disease [4]. However, inappropriate cognition and poor adherence make these approaches impossible for many people in real-world settings [5]. Therefore, exploring convenient and effective adjunctive therapies with minimal side effects is warranted.

Acupuncture has a long history of treating diseases. It is increasingly accepted by patients in many parts of the world, as accumulating high-level evidence on the effect and safety of acupuncture is recognized [6–8]. For NAFLD, a meta-analysis including eight randomized controlled trials (RCTs) with 939 patients showed that acupuncture or acupuncture plus conventional medicine was superior to conventional medicine alone in terms of overall clinical efficacy, mainly including biochemical test [9]. Another meta-analysis of acupoint therapy, including 3 RCTs with 108 patients, showed that the effectiveness of EA in decreasing TC and TG was significantly changed than manual acupuncture because of the higher stimulation intensity of EA [10]. However, no well-designed studies compared the efficacy of acupuncture versus placebo/sham acupuncture on variables measured by internationally accepted modalities and chose the internationally recognized measurement as the outcome [11].

Therefore, we conduct this single-center, randomized, sham-acupuncture controlled, patients-blinded pilot study to investigate the efficacy and safety of electro-acupuncture on the reduction of relative liver fat content measured by MRI-PDFF in NASH patients.

## Patients and methods

### Study design

This single-center, randomized, sham-acupuncture controlled trial was conducted between June 2021 and July 2022. The protocol was approved by the institutional review board and ethics committee of Beijing Friendship Hospital, Capital Medical University (No. 2021-P2-071-03). The study was registered with ChiCTR (www.chictr.org.cn) on 23 May 2021 before recruiting the first patient (ChiCTR2100046617). Before participation, all patients provided written informed consent. The results of this study are reported to be consistent with the Additional file 7: CONSORT 2010 checklist.

### Patient selection

The study population included men and women between the ages of 18 and 65 years if they had a diagnosis of NASH based on either A) a liver biopsy within 12 months of screening which was consistent with NASH with fibrosis (no cirrhosis) or B) phenotypic diagnosis: a) fatty liver diagnosed by ultrasound or CT scan or vibration-controlled elastography (VCTE, with FibroScan or FibroTouch), b) ALT $\ge$ 1.5 $\times$ ULN confirmed two times with 1 week apart in the previous 3 months, c) BMI $\ge$ 25 kg/m^2^, d) baseline MRI-PDFF showing $\ge$ 8% steatosis.

Critical exclusion criteria included hepatitis B, C, and other liver diseases, advanced liver cirrhosis, decompensation, and evidence of HCC. Patients receiving acupuncture within 1 month before enrollment were also excluded. The supplement provided a complete list of the inclusion and exclusion criteria (Additional file 1: Table S1).

### Randomization and blinding

Eligible participants were randomized in a 1:1 ratio to receive SA or EA. Block randomization was used in this trial, and the block size was 6. A biostatistician who did not participate in this trial created a randomization sequence. The random number was assigned after the participants had met all inclusion criteria and completed baseline assessments. Acupuncturists were not blinded to the treatment.

Patients, outcome assessors, and statisticians were blinded to group assignment. These two groups were identified as groups 1 and 2 during the statistical analysis.

### Intervention (SA or EA)

The therapeutic regimen was based on the TCM theory, and the clinical experience of the research assistant used a centralized randomization procedure to implement the allocation schedule expert acupuncturist. All licensed acupuncturists involved in this trial had at least 3 years of experience in acupuncture. Before patient enrollment, all acupuncturists participated in the training of standardized operating procedures, including the location of acupoints and non-acupoints and the manipulation of needling. Sterile disposable needles (length 40–60 mm, diameter 0.3 mm; HuanQiu, Suzhou, China) were used. A 30-min treatment was delivered thrice weekly for 12 weeks (usually every other day) with 36 sessions. All participants received education about eating and physical activity according to guidelines from the American Gastroenterological Association (AGA) institute clinical practice updates Committee [5] before treatment, and they did not allow to accept combining the other method of traditional Chinese medicine, such as decoction, acupoint catgut embedding and so on.

Patients in the SA group received superficial skin penetration (2 to 3 mm in depth) at non-acupoints was done without “de qi” manipulations. The location of non-acupoints was shown in Additional file 1: Table S2 and Additional file 2: Fig. S1, parts of points referred to by previous study [6]. Then paired electrodes from the Hwato electro-acupuncture instrument (SDZ-II, Suzhou Medical Co, Ltd, Jiangsu, China) were used at NA2-NA3, but no electrical stimulus was applied.

Patients in the EA group received needling at traditional acupuncture points, including CV12 (Zhongwan), CV4 (Guanyuan), bilateral ST25 (Tianshu), SP15 (Daheng), LV13 (Zhangmen), ST36 (Zusanli), SP6 (Sanyinjiao), LI4 (Hegu), and LV3 (Taichong). The location of points was described in the Additional file 1: Table S3 and Additional file 3: Fig. S2. Following insertion, needles were manipulated by twirling, lifting, and thrusting for 30 s at each acupoint to achieve “de qi” (a composite of sensations including soreness, numbness, distention, and heaviness), which was believed to be an essential component for acupuncture’s efficacy. Then paired electrodes from the same instrument were used at bilateral ST25-SP15 by the acupuncturists. The electro-acupuncture lasted for 30 min with a dilatational wave of 2 Hz and an intensity of 0.1 to 1 mA depending on the participant’s comfort level.

Similarities and differences between the two acupuncture groups were shown in the Additional file 1: Table S4 and Additional file 4: Fig. S3.

### Outcomes

The primary outcome was the changes in relative liver fat content measured by MRI-PDFF [12] at week 12. Critical secondary endpoints included the patients with a 30% relative decline in MRI-PDFF, the change of liver stiffness by MRE at weeks 12 and 16, and the change of biochemical variables at week 12.

All patients underwent non-contrast scans using a 3.0-Tesla MRI scanner (MR750, GE Healthcare, Waukesha, WI, USA) with an eight-channel phased-array body coil centered over the liver. The whole liver was covered during the axial IDEAL IQ examination. PDFF measured by locations included each of the Couinaud segments of the liver (including S1 through S8). The area of Couinaud components showed in Additional file 5: Fig. S4. Detailed Standard Operating Procedure (SOP) of MRI-PDFF can be found in our recent publication [13]. MRE measurement used a two-dimensional (2D) spin-echo–echo planar imaging (SE- EPI) MRE sequence; detailed Standard Operating Procedure (SOP) of MRE can be found in our group publication [14] (Additional file 6: Fig. S5).

Exploratory outcomes including the score of several questionaries about sleep quality assessment (questionnaire of Athens insomnia scale-8, AIS-8), emotional assessment (patient health questionnaire-2, PHQ-2 and generalized anxiety disorder-2, GAD-2), quality of life (chronic liver disease questionnaire, CLDQ-NAFLD/NASH) [15], the behavior of eating (the three-factor eating questionnaire-21, TFEQ-21) [16], physical activity (international physical activity questionnaire-7, IPAQ-7) [17] and attitude of behavior change (the university of Rhode Island change assessment scale URICA) [18].

A complete procedure of enrollment, intervention, and assessments of this study was provided in Additional file 1: Table S5.

### Adverse events

Participants or acupuncturists monitored and recorded acupuncture-related and unrelated adverse events (AEs). Common acupuncture-related AEs included subcutaneous hematoma, residual needling sensation after needle removal, itching at the sites of needle insertion, and dizziness throughout the treatment.

### Considerations for sample size calculation

This is a pilot study; the minimum sample size for exploratory trials is 20 to 30 per group, according to Provisions for Drug Registration in China. It is generally accepted that at least 30 participants are required for a pilot study [19]. Based on clinical experience and published references of pilot study [20, 21], a total sample size of 60 subjects was needed to ensure that both SA and EA groups. The results of this study were intended to facilitate the calculation of the appropriate sample size for a further, adequately powered randomized clinical trial.

### Statistical methods

Descriptive analyses were performed on all baseline variables. Continuous variables whose distribution met normality assumptions were given as mean ± standard deviation or else given as medians and quartiles (quartile 1 [Q1], quartile 3 [Q3]); categorical variables were presented as the absolute value and relative frequency. The comparisons of continuous or categorical variables were examined using the t-test, non-parametric Mann–Whitney test, or Chi-square test, as appropriate.

The outcomes were analyzed according to the intention-to-treat (ITT) principle and using imputed data to avoid potential confounding by missing data. All randomized participants were included in the ITT analysis. Assuming that the occurrence of missing data was random, we used multiple imputations by chained equations to impute one complete dataset with five interactions. The imputation models included all variables under analyses [22].

The sensitivity analysis was performed in the per-protocol (PP) set; the PP set was used for all participants who completed the treatment ($\ge$ 80% completed sessions) and follow-up without significant violations.

The analyses were implemented with SAS version 9.4 (SAS institute Inc Cary, NC) and R version 4.1.2 (https://www.r-project.org/). P < 0.05 was considered statistically significant.

## Results

### Baseline characteristics of the enrolled patients

**Fig. 1:**
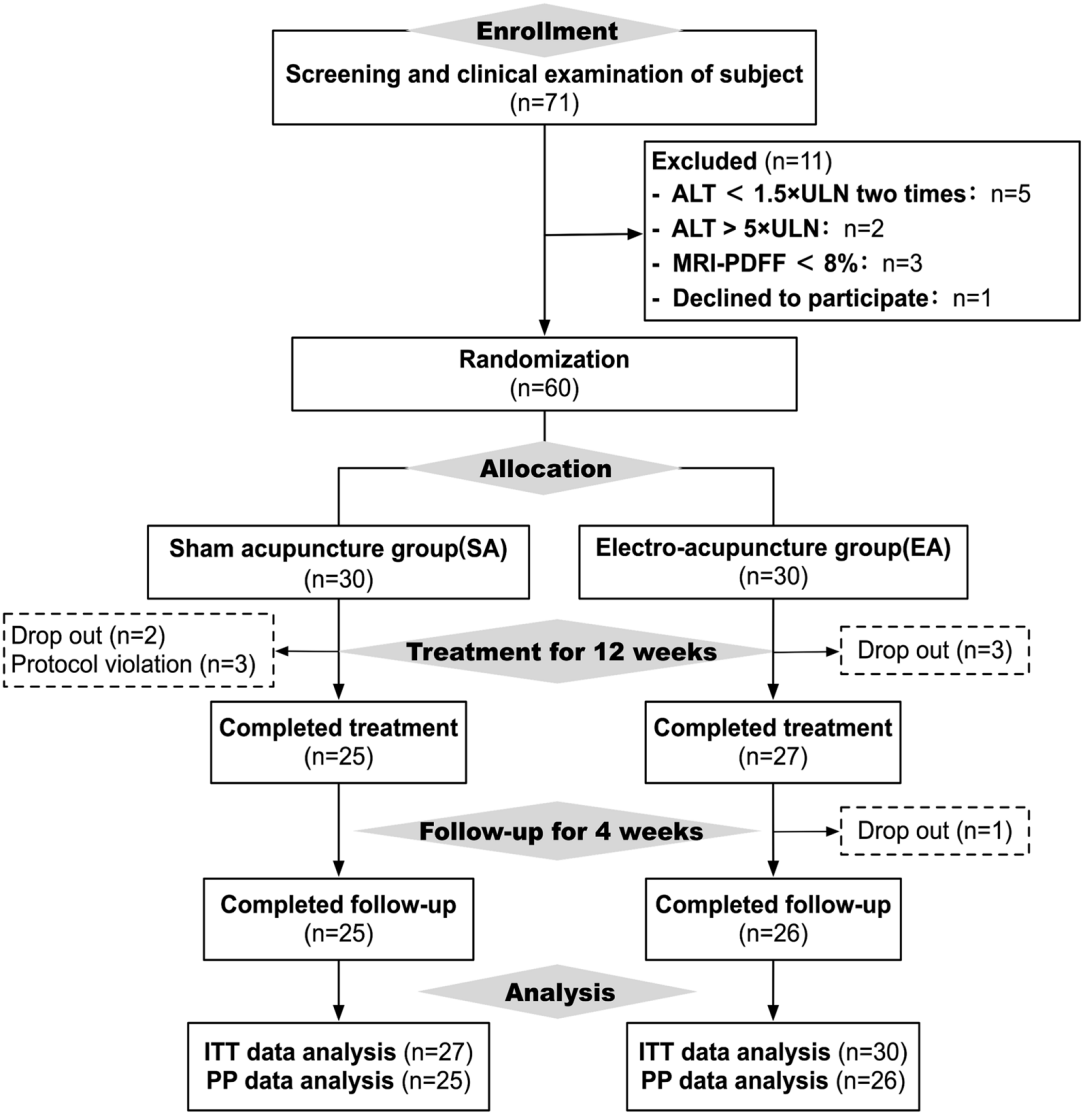
Flow chart of the study from enrollment to analysis

Between May 2021 and July 2022, 71 consecutive patients with NASH were screened. Of these, 11 (15.5%) were excluded for various reasons. Consequently, 60 patients were recruited. A total of 6 (10%) patients dropped out of the study (4 in the electro-acupuncture group and 2 in the sham acupuncture group; 5 during the 12-week treatment and one during the 4-week follow-up; 4 patients had home quarantine for relative to COVID-19 and two patients had no time to treatment for on business.) (Fig. 1).

Of the patients who completed the 12-week treatment, 25 were in the sham acupuncture group, and 26 were in the electro-acupuncture group. Unfortunately, three patients were protocol violations in the sham acupuncture group because the ALT > 5 $\times$ ULN in the screening period and the investigator missed. In the process of trial, the drugs used by recorded strictly, two groups had no significant differences (Additional file 1: Table S6).

Patient demographic characteristics were similar between the two groups at baseline, except the gender of female in the electro-acupuncture group [22/30 (73.3%)], compared to the sham-acupuncture group [12/27 (44.4%)]; these differences turned out to be statistically significant (*p* = 0.026*) (Table 1). The average age was 41.6 ± 10.8 years in the EA group and 39.1 ± 10.8 in the SA group; in two groups of patients with smoking, drinking, type 2 diabetes, and hypertension, the mean baseline values for liver fat content by MRI-PDFF, liver stiffness by MRE, biochemical varieties by blood test and anthropometry parameters were no significant difference (*p* > 0.05).

**Table 1:** Baseline characteristics of patients in SA and EA group

	Sham acupuncture (SA) n = 27	Electro-acupuncture (EA) n = 30	*p* value
Age, years (mean $\pm$ SD)	41.6 $\pm$ 10.8	39.1 $\pm$ 10.8	0.389
Gender, n (%)			0.026*
Female	12 (44.4%)	22 (73.3%)	
Male	15 (55.6%)	8 (26.7%)	
Smoking, n (%)	3 (11.1%)	3 (10.0%)	0.891
Drinking, n (%)	5 (18.5%)	6 (20.0%)	0.888
Diabetes, n (%)	5 (18.5%)	9 (30.0%)	0.315
Hypertension, n (%)	9 (33.3%)	9 (30.0%)	0.787
Non-invasive Test			
Liver fat by MRI-PDFF, % [medium (p25, p75)]	20.8 (14.0, 28.1)	20.3 (13.8, 25.1)	0.371
Liver stiffness by MRE, kPa (mean $\pm$ SD)	2.4 $\pm$ 0.7	2.6 $\pm$ 0.9	0.519
Anthropometry parameters			
BMI, kg/m^2^ (mean $\pm$ SD)	31.8 $\pm$ 4.9	33.0 $\pm$ 4.1	0.342
Body weight, Kg (mean $\pm$ SD)	91.4 $\pm$ 19.3	90.9 $\pm$ 14.0	0.899
WHR % (mean $\pm$ SD)	92.8 $\pm$ 6.7	92.5 $\pm$ 5.6	0.870
Biochemical varieties			
ALT, U/L [medium (p25, p75)]	89.0 (81.5, 114.0)	95.5 (78.0, 121.0)	0.576
AST, U/L [medium (p25, p75)]	50.5 (44.5, 66.0)	60.6 (48.9, 80.1)	0.099
GGT, U/L [medium (p25, p75)]	67.0 (40.0, 78.0)	54.0 (37.8, 79.8)	0.533
ALP, U/L (mean $\pm$ SD)	92.8 $\pm$ 27.5	89.8 $\pm$ 23.8	0.659
TC, mmol/L (mean $\pm$ SD)	5.7 $\pm$ 1.0	5.6 $\pm$ 1.3	0.675
TG, mmol/L [medium (p25, p75)]	1.8 (1.3, 2.2)	1.7 (1.4, 3.4)	0.743
LDL-C, mmol/L (mean $\pm$ SD)	3.5 $\pm$ 0.7	3.3 $\pm$ 0.7	0.296
FBG, mmol/L (mean $\pm$ SD)	5.9 $\pm$ 1.1	6.1 $\pm$ 1.2	0.522
HbA1c % (mean $\pm$ SD)	5.9 $\pm$ 0.8	6.00 $\pm$ 0.7	0.624
HOMA-IR [medium (p25, p75)]	6.9 (4.3, 13.5)	9.3 (5.6, 13.7)	0.174

### Relative liver fat content by MRI-PDFF was significantly decreased in the EA group

The primary endpoint was the relative change of liver content by MRI-PDFF from baseline to 12 weeks in this study. The MRI-PDFF reduced rapidly in 12 weeks of treatment by EA and maintained a reduction trend during the next 4 weeks in follow-up. However, the liver fat reduction was slow in the SA group at 12 weeks and had a slight upward trend in 4 weeks in follow-up.

**Fig. 2:**
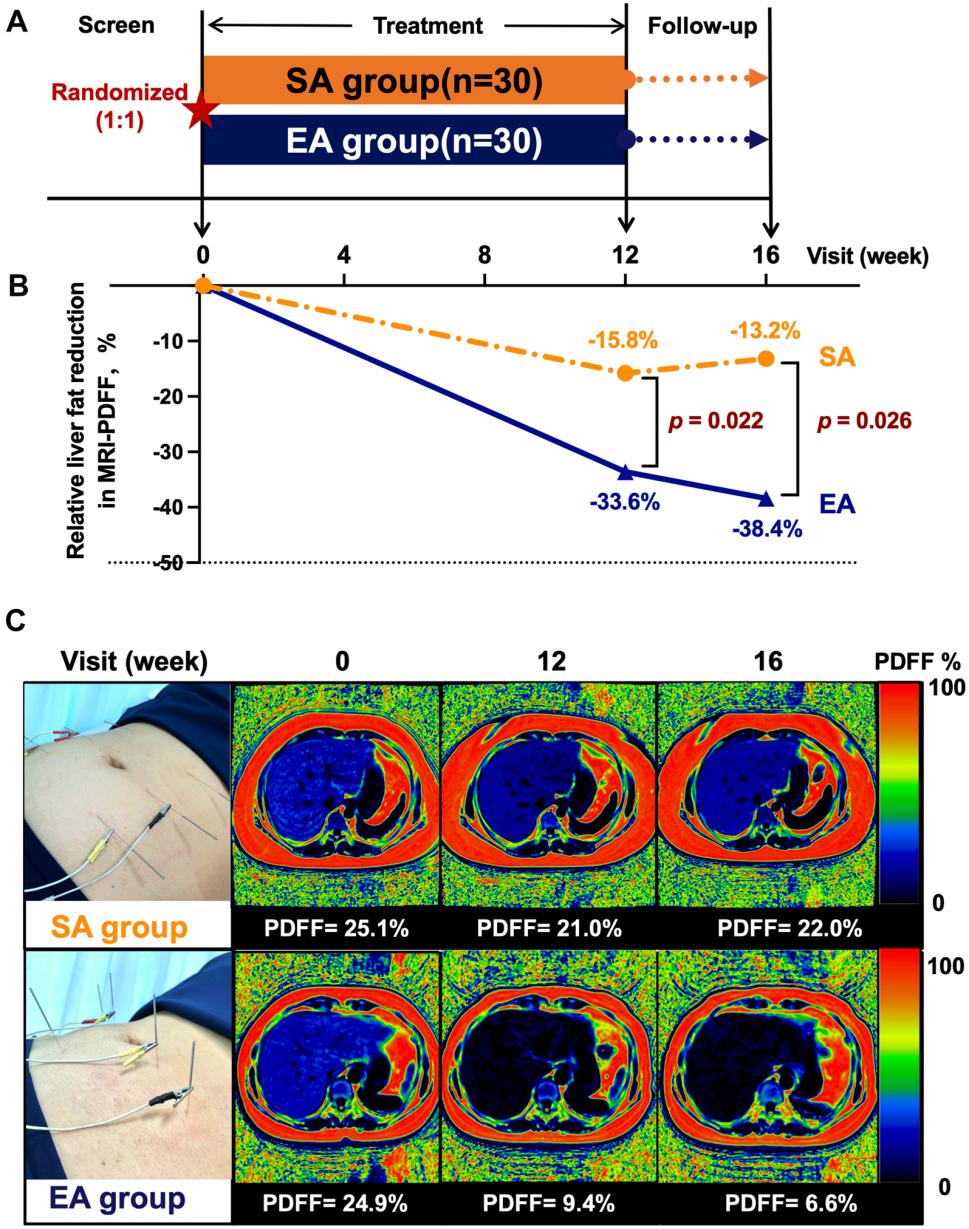
The clinical study design and primary outcome of MRI-PDFF reduction in the SA and EA group. **A** The procedure of this trial was from screen to follow-up in the SA (orange color) and EA (blue color) groups. **B** The primary outcome in the SA (orange color) and EA (blue color) groups was after 12 weeks of treatment and 4 weeks of follow-up. **C** Representative case from the SA and the EA group by MRI-PDFF. SA: sham-acupuncture; EA: electro-acupuncture; PDFF: proton density fat fraction

In the ITT population, the relative liver fat reduction from baseline in MRI-PDFF at week 12 (the primary endpoint) was − 15.80% (− 36.1%, − 2.7%) in the SA group, − 33.6% (− 52.9%, − 22.7%) in the EA group, two groups have statistically significant (*p* = 0.022*). The relative liver fat reduction from baseline in MRI-PDFF at week 16 was − 13.2% (− 35.0%, − 3.2%) in the SA group, − 38.4% (− 55.3%, − 16.6%) in the EA group, two groups have statistically significant (*p* = 0.026*) (Table 2, Fig. 2B). Based on the unbalance of gender in the two groups, we used the linear regression analyses with the primary endpoint to exclude the potential confounding effect of gender (Additional file 1: Table S7). In the PP population, the result was like ITT (Additional file 1: Table S8).

**Table 2:** Treatment outcomes of patients in the SA and EA group

	Sham acupuncture (SA) n = 27	Electro-acupuncture (EA) n = 30	*p* value
*Primary Endpoint*			
Relative liver fat reduction from baseline in MRI-PDFF %, [medium (p25, p75)]			
Week 12	− 15.8 (− 36.1, − 2.7)	− 33.6 (− 52.9, − 22.7)	0.022*
Week 16	− 13.2 (− 35.0, − 3.2)	− 38.4 (− 55.3, − 16.6)	0.026*
*Secondary endpoints*			
Patients with a ≥ 30% relative decline in MRI-PDFF%, n (%)			
Week 12	7/27 (25.9%)	16/30 (53.3%)	0.035*
Week 16	11/27 (40.7%)	18/30 (60.0%)	0.146
Liver stiffness by MRE, kPa [medium (p25, p75)]			
Week 12	0.2 (− 0.2, 0.6)	− 0.1 (− 0.5, 0.3)	0.156
Week 16	0.2 (− 0.2, 0.5)	− 0.1 (− 0.4, 0.4)	0.201
BMI, kg/m^2^ [medium (p25, p75)]			
Week 12	− 0.6 (− 1.4, 0.3)	− 1.3 (− 3.0, − 0.3)	0.024*
Week 16	− 0.2 (− 1.2, 0.3)	− 1.5 (− 2.4, − 0.3)	0.013*
Body weight, kg [medium (p25, p75)]			
Week 12	− 1.3 (− 4.7, 0.5)	− 3.0 (− 6.4, − 0.5)	0.185
Week 16	0.1 (− 2.9, 0.9)	− 3.0 (− 6.0, − 0.8)	0.034*
WHR, % [medium (p25, p75)]			
Week 12	− 0.7 (− 5.8, 2.3)	− 2.3 (− 4.7, 1.0)	0.260
Week 16	− 0.3 (− 4.0, 1.8)	− 0.8 (− 4.3, 2.8)	0.528
Biochemical varieties, Week 12 [medium (p25, p75)]			
ALT, U/L	− 34.0 (-49.0, − 10.0)	− 52.5 (− 76.0, − 20.0)	0.114
AST, U/L	− 16.2 (− 24.3, − 6.4)	− 27.4 (− 45.1, − 15.0)	0.015*
GGT, U/L	− 15.0 (− 26.0, − 7.0)	− 13.0 (− 28.0, − 5.0)	0.749
ALP, U/L	0.0 (− 11.0, 6.0)	− 0.5 (− 5.0, 12.0)	0.667
CHO, mmol/L	0.0 (− 0.7, 0.6)	− 0.3 (− 1.0, 0.2)	0.228
TG, mmol/L	− 0.3 (− 0.9, 0.2)	− 0.5 (− 0.9, 0.1)	0.306
LDL, mmol/L	− 0.1 (− 0.5, 0.3)	− 0.1 (− 0.4, 0.3)	0.848
FBG, mmol/L	− 0.3 (− 0.4, 0.2)	− 0.3 (− 0.8, 0.0)	0.139
HbA1c%	0.0 (− 0.3, 0.1)	− 0.2 (− 0.3, 0.0)	0.161
HOMA-IR	− 1.6 (− 3.9, 0.4)	− 2.2 (− 5.7, − 0.7)	0.164

The number of patients who achieved a 30% or more relative reduction of liver fat content at week 12 was 7 (25.9%) of 27 patients in the SA group, 16 (53.3%) of 30 patients in the EA group; there were significant differences (*p* = 0.035*) (Table 2). At week 16, 11 (40.7%) of 27 patients in the SA group and 18 (60.0%) of 30 patients in the EA group, there were no significant differences (*p* = 0.146) (Table 2). In the PP population, there were significant differences in the two groups at weeks 12 and 16 (*p* = 0.016* and 0.036*) (Additional file 1: Table S8).

### Changes in anthropometry parameters in the EA group

In the ITT population, the change in BMI from baseline to weeks 12 and 16 were − 0.6 (− 1.4, 0.3) and − 0.2 (− 0.2, 0.3) in the SA group, − 1.3 (− 3.0, − 0.3) and − 1.5 (− 2.4, − 0.3) in the EA group, which was statistically significant (*p* = 0.024* and 0.013*).

In the change of body weight from baseline to week 12 in the two groups, there was no significant difference (*p* = 0.185), but EA lost more weight [− 3.0 (− 6.0, − 0.8)] than SA [0.1 (− 2.9, 0.9)] at week 16 (*p* = 0.034*) (Table 2).

The change of WHR from baseline to week 12 and week 16 in the two groups have little significant difference (*p* = 0.260 and 0.528); however, the more reduction trend in the EA group [− 2.3 (− 4.7, 1.0) and − 0.8 (− 4.3, 2.8)] than SA group [− 0.7 (− 5.8, 2.3) and − 0.3 (− 4.0, 1.8)] at weeks 12 and 16. (Table 2).

### Serum AST significantly declined in the EA group.

In the ITT population, the change of AST from baseline to week 12 was − 16.2 (− 24.3, − 6.4) in the SA group, − 27.4 (− 45.1, − 15.0) in the EA group, there were significant differences (*p* = 0.015*), the change of biochemical variables by a blood test from baseline to week 12 in the EA group compared to the SA group, which was not statistically significant (Table 2). In the PP population, the result was similar to ITT (Additional file 1: Table S9).

### Liver stiffness by MRE had no significant change in either group

In the ITT population, the change of liver stiffness from baseline in MRE at week 12 and week 16 was [− 0.1 (− 0.5, 0.3) and − 0.1 (− 0.4, 0.4)] in the EA group, [0.2 (− 0.2, 0.6) and 0.2 (− 0.2, 0.5)] in the SA group, two groups had not statistically significant (*p* = 0.156) (Table 2). The PP population's result was similar to ITT (Additional file 1: Table S9).

### Behavior and emotional questionaries characteristics in two groups

**Fig. 3:**
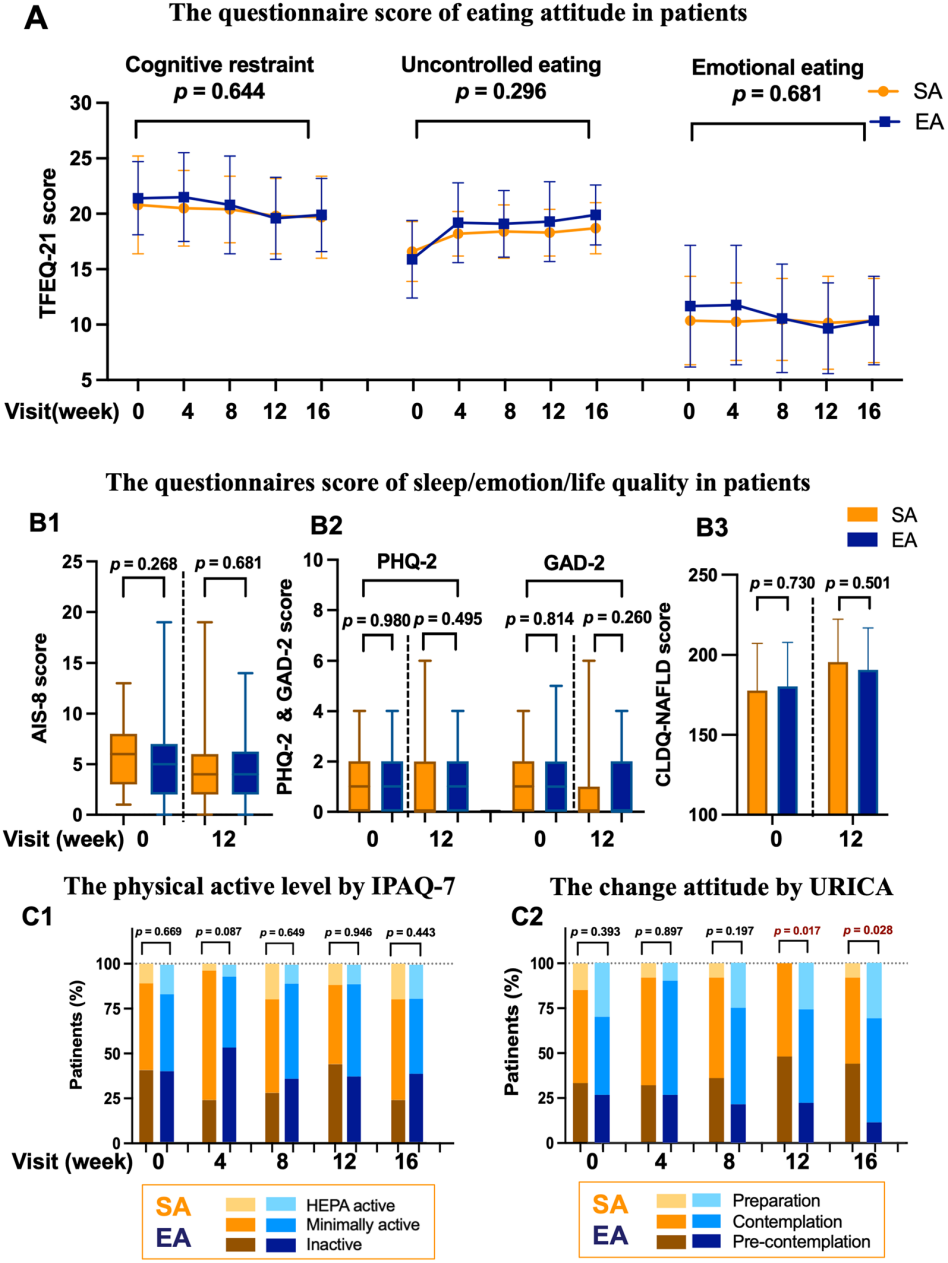
Behavior and emotional questionaries characteristics in the SA and EA group. **A** The questionnaire score of eating attitude by TEFQ-21 in the SA (orange color) and the EA (blue color) groups from the baseline to week 16. There were three factors of eating behavior: cognitive restraint, uncontrolled eating, and emotional eating. **B** The questionnaire score of sleep/emotion/life quality in the SA (orange color) and EA (blue color) groups. **B1** The score of AIS-8 shows the quality of sleep. **B2** The score of PHQ-2 and GAD-2 reflects depression and anxiety emotion. **B3** The score of CLDQ-NAFLD/NASH shows the quality of life in NASH patients. **C1** The score of IPAQ-7 reflects the physical activity level in the SA (orange color series) and EA (blue color series) groups; more light color showed HEPA active (≥ 3000 MET-minutes/week), the medium color showed minimally active (600–2999 MET-minutes/week), the more dark color led inactive (≤ 599 MET-minutes/week). **C2** The score of URICA to reflect the physical activity level in the SA (orange color series) and EA (blue color series) groups, the patients in the preparation period (more light color), in the contemplation period (medium color), in pre-contemplation period (more dark color). TEFQ-21: the three-factor eating questionnaire-21; AIS-8: questionnaire of Athens insomnia scale-8; PHQ-2: patient health questionnaire-2; GAD-2: generalized anxiety disorder-2; CLDQ-NAFLD/NASH: chronic liver disease questionnaire for NAFLD/NASH; IPAQ-7: international physical activity questionnaire-7; HEPA: health-enhancing physical activity; MET: metabolic equivalent; URICA: the university of Rhode Island change assessment scale

In the ITT population, the characteristics of three factors of eating attitude included cognitive restriction, uncontrolled eating, and emotional eating by TFEQ-21 at baseline, week 4, week 8, week 12 to week 16 was no significant difference between the two groups (Fig. 3A). The sleep quality (Fig. 3B1) and depression, anxiety assessments (Fig. 3B2), and quality of life for NAFLD/NASH (Fig. 3B3) were not a significant difference between the two groups at baseline and week 12. The characteristics of physical activity level by IPAQ-7 at baseline, week 4, week 8, week 12 to week 16 were not significantly different between the two groups (Fig. 3C1).

The attitude of change behavior assessment by URICA at baseline, week 4, and week 8 were similar between the two groups. However, the number of patients who were in the preparation period at week 12 and week 16 was 7 (25.9%) of 27 patients and 8 (30.8%) of 26 patients in the EA group, 0 (0.0%), and 2 (8.0%) of 25 patients in the SA group, and there were significant differences (*p* = 0.017* and 0.028*) (Fig. 3C2).

### Blind assessment and credibility/expectancy questionnaire in patients

Patients were unaware of assigned treatments after the first treatment in two groups (*p* = 0.697). However, unsuccessful blinding was maintained after the last treatment (week 12), and there were significant differences between the two groups (*p* = 0.000*) (Additional file 1: Table S10).

Patients with the credibility/expectancy score after the first treatment and after the last treatment in the two groups had no significant differences (*p* = 0.725 and 0.174) (Additional file 1: Table S11).

### Adverse events

Adverse events related to acupuncture were reported in 3 (11.11%) of 27 in the SA group and 2 (6.67%) of 30 patients in the EA group. The most frequently reported adverse events included hematoma and residual needling sensation after needle removal. All events were mild and self-limiting, and none required medical interventions. There were no serious adverse events (Table 3).

**Table 3:** Adverse Events during treatment in the SA and EA group

	Sham acupuncture (SA) n = 27	Electro-acupuncture (EA) n = 30	*p* value
AEs related to acupuncture, no. (%)			
Serious AEs	0 (0.0%)	0 (0.0%)	–
Subcutaneous Hematoma	3 (11.1%)	1 (3.3%)	0.251
Post-needling pain	0 (0.0%)	1 (3.3%)	0.399
AEs of unrelated to acupuncture, no. (%)			
Cold and cough	6 (22.2%)	6 (20.0%)	0.837
Dental ulcer	1 (3.7%)	0 (0.0%)	0.288
Toothache	1 (3.7%)	0 (0.0%)	0.288
Headache	1 (3.7%)	1 (3.3%)	0.940
Insomnia	2 (7.4%)	0 (0.0%)	0.129
Stomached	0 (0.0%)	2 (6.7%)	0.172
Diarrhea	2 (7.4%)	0 (0.0%)	0.129
Constipation	0 (0.0%)	1 (3.3%)	0.399
U-arthritis	0 (0.0%)	1 (3.3%)	0.399
Paramenia	1 (3.7%)	0 (0.0%)	0.288
Shingles	1 (3.7%)	0 (0.0%)	0.288
Total number of AEs, no. (%)			
	18	13	–

Adverse events of unrelated to acupuncture, including cold, dental ulcer, toothache, insomnia, stomach, diarrhea, constipation, U-arthritis, paramecia, and shingles, had not a significant difference (*p* > 0.05) (Table 3).

## Discussion

In this prospective randomized sham-acupuncture controlled, patient-blinded trial, we demonstrated that 12 weeks of EA treatment significantly reduced the relative liver fat content, with patients achieving a ≥ 30% decline in MRI-PDFF. The therapeutic benefits were maintained and continued for at least 4 weeks after completion of the treatment. In many cases, acupuncture plays a variety of influential roles in the treatment of NAFLD through the intervention of multiple targets and multiple signaling pathways, such as by inhibiting the inflammatory response, regulating lipid metabolism disorder, antagonizing oxidative stress injury, and interfering with endoplasm reticulum stress, among others [23]. Notably, the better effective and specific mechanism of electro-acupuncture was revealed by anti-inflammation [24], lipid-lowering [25], and mitigating insulin resistance [26]. It provides potential mechanisms of electro-acupuncture intervention in NASH.

In the theory of Chinese medicine, the pathogenesis of NAFLD is based on the “deficiency of the spleen and stagnation of the liver and Qi.” We choose Zhongwan (CV12), Tianshu(ST25), and Daheng(SP15) have the effect of regulating gastrointestinal activities [6]. Zusanli(ST36) combined with Sanyinjiao(SP6) can support the function of spleen and stomach transportation and transfusion [27]. Zhangmen (LV13), Hegu (LI4), and Taichong (LV3) can smooth the liver qi to promote digestion and regulate endocrine. Guanyuan (CV4) is the main acupoint for strengthening the power of immunity and metabolism following the theory of Chinese medicine [28]. Above acupoint stimulation can help suppress appetite, accelerate glucose and lipid metabolism, and anti-inflammation in NASH.

To our knowledge, this study is the first randomized controlled trial testing the efficacy of EA in NASH patients by using MRI-PDFF. Compared with other imaging modalities, MRI-PDFF has the highest diagnostic accuracy for quantifying liver fat content and is commonly used in trials in NASH [29]. The FDA, in December 2018, issued guidance for clinical trial design for patients with noncirrhotic NASH, permitting the use of MRI-PDFF as the method for enrolment and evaluating therapeutic effect for early-phase clinical trials in NASH instead of liver biopsy [30]. In patients with NAFLD, change in MRI-PDFF (≥ 30% decline relative to baseline) is associated with a histological response (NAFLD activity score ≥ 2 points improvement with no worsening of fibrosis) and fibrosis regression (reduction of one or more stages) [31]. Therefore, our study would be a perfect jumping point for further validating the therapeutic efficacy of EA for NASH.

Indeed, previous studies have shown that acupuncture or EA can reduce body weight and improve NAFLD [32–34]. However, none of the studies included in a meta-analysis adopted sham acupuncture as a control, and none used MRI-PDFF as the study endpoint [9]. In the current study, we used the SA with non-acupoint and superficial insert method, hoping to decrease the chance for the participants to discriminate between real EA and SA [35]. However, we still found that the reduction of relative liver content by MRI-PDFF was − 15.8% in the SA group, which is higher than the placebo response (− 2.4%) in trials for new therapeutic agents [36]. These facts indicated the necessity to have a SA as a control to avoid overestimation of the therapeutic efficacy of EA.

Our study also demonstrated an effective reduction in body weight and serum AST, which is in line with a previous survey [32]. However, except for serum AST, the other biological markers, including ALT, GGT, TC, TG, and FBG, decreased have no significant change compared with the SA group. We assumed the reasons for the lack of statistical significance include the short treatment duration period and the relatively small sample size.

Our study also has several limitations. First, the present study was a single-center pilot study on a small number of patients for a relatively short period. Second, not all patients with histological diagnosis of NASH, but we hope that as a proof-of-concept stage in clinical trials, the use of well-accepted MRI-PDFF to evaluate the effect of liver fat content would be reasonable [12]. Third, the blind patients were unsuccessful since 80.0% of the SA group correctly guessed their treatment allocation at week 12. However, we hope that using objective imaging and biochemical endpoints could partially counter this downside.

In conclusion, this randomized, SA-controlled pilot study of twelve weeks of EA could effectively reduce the liver fat content measured by MRI-PDFF. Further multiple centers participate in blinded, SA-controlled studies are justified to verify the therapeutic efficacy of electro-acupuncture for NASH patients.

## Supplementary Information

**Additional file 1:** Supplementary tables.

**Additional file 2:** Locations of non-acupoints in sham acupuncture(SA) group.

**Additional file 3:** Locations of acupoints in electro-acupuncture(EA) group.

**Additional file 4:** The real image of different acupuncture method in two groups.

**Additional file 5:** Quality control of MRI-PDFF.

**Additional file 6:** Quality control of MRE.

**Additional file 7:** Consort checklist.

## Data Availability

The datasets used and analyzed during the current study are available from the corresponding author upon reasonable request.

## Abbreviations

NASH: Non-alcoholic steatohepatitis
NAFLD: Non-alcoholic fatty liver disease
HCC: Hepatocellular carcinoma
EA: Electro-acupuncture
SA: Sham-acupuncture
MRI-PDFF: Magnetic resonance imaging proton density fat fraction
MRE: Magnetic resonance elastography
ALT: Alanine transferase
AST: Aspartate transferase
GGT: γ-Glutamyl transferase
ALP: Alkaline phosphatase
TC: Total cholesterol
TG: Triglyceride
LDL-C: Low density lipoprotein cholesterol
FBG: Fasting blood glucose
INS: Fasting insulin
HbA1c%: Glycosylated hemoglobin
HOMA-IR: Homeostatic model assessment insulin resistance
BMI: Body mass index
WHR: Waist hip rate
AIS-8: Questionnaire of Athens insomnia scale-8
PHQ-2: Patient health questionnaire-2
GAD-2: Generalized anxiety disorder-2
CLDQ- NAFLD/NASH: Disease-specific quality of life via the chronic liver disease questionnaire
TFEQ-21: The three-factor eating questionnaire-21
IPAQ-7: International physical activity questionnaire-7
URICA: The university of Rhode Island change assessment scale
