# Supplementary material for: Electro-acupuncture reduced steatosis on MRI-PDFF in patients with non-alcoholic steatohepatitis: a randomized controlled pilot clinical trial
Source: Chin Med. 2023 Feb 24;18:19. doi: 10.1186/s13020-023-00724-w (PMC9950708; doi:10.1186/s13020-023-00724-w)
Supplement: Supplementary file 1 — Additional file 1: Supplementary tables. [file 13020_2023_724_MOESM1_ESM.docx]

**Table S1. Inclusion and Exclusion criteria in the study**

|  | | **Criteria** | **Selection** |
| --- | --- | --- | --- |
| Inclusion criteria (Participants must fulfill all the following criteria) | | |  |
| 1 | Male and female patients 18 to 65 years age (including 18 and 65 years old). | | require |
| 2 | Imaging shows fatty liver (B-ultrasound or VCTE or CT or MRI). | | require |
| 3 | NASH diagnosis by biopsy or clinical features:  A. NASH diagnosis by biopsy: histological evidence of NASH by liver biopsy within 6 months before screening or during the screening period (NAS score ≥ 4 points, including inflammation and ballooning at least 1 point each, and fibrosis level ≤ F3), No other chronic liver diseases were found in histological diagnosis.  B. NASH diagnosis by clinical features: ALT≥ 1.5×ULN, two examinations with an interval greater than 7 days within 3 months and BMI ≥ 25 kg/m^2^ and liver fat ≥ 8% by MRI-PDFF at baseline. | | A or B |
| 4 | Sign the informed consent. | | require |
| Exclusion criteria (Participants mustn't be eligible for any of the following criteria) | | |  |
| 1 | History of other hepatobiliary diseases, including but not limited to hepatitis B or C virus infection, chronic alcoholic liver disease, drug-induced liver disease, autoimmune hepatitis, primary biliary liver Sclerosis, primary sclerosing cholangitis, Wilson ‘s disease, α1-antitrypsin deficiency, liver cancer, or obvious liver function abnormalities (ALT or AST ≥ 5×ULN), etc. | | require |
| 2 | Excessive drinking for 3 consecutive months or more in the 1 year before screening (the average daily consumption of ethanol for men is more than 30 grams, which is equivalent to 3.75 units of alcohol, and for women, more than 20 grams, which is equivalent to 2.5 units of alcohol: 1 unit = 285 mL of beer, or strong 25 mL wine, or 100 mL wine). | | require |
| 3 | Liver biopsy indicates liver cirrhosis or a previous clinical diagnosis of liver cirrhosis. | | require |
| 4 | The history of bariatric surgery or the plan to bariatric surgery soon. | | require |
| 5 | Type 1 diabetes patients and uncontrolled type 2 diabetes patients (HbA1c ≥ 9.5%). | | require |
| 6 | Other diseases that are unstable or untreated (including but not limited to the gastrointestinal tract, nerve, blood, endocrine, tumor, lung, immune, mental diseases). | | require |
| 7 | History of acute cardiovascular events, or a history of the following cardiovascular and cerebrovascular diseases: coronary angioplasty, stroke, transient ischemic attack, coronary heart disease. | | require |
| 8 | Taking drugs that may cause steatosis/steatohepatitis (including amoxicillin, methotrexate, hormones, tetracycline, tamoxifen, steroids, valproic acid, etc.). | | require |
| 9 | Taking hypoglycemic drugs GLP-1, thiazolidinedione insulin sensitizer-related drugs, weight loss drugs (including but not limited to sibutramine, orlistat) and clinically shown to improve steatohepatitis (including but not limited to liraglutide, obeticholic acid, Elafibranor, vitamin E, etc.) 3 months before randomization. | | require |
| 10 | Patients who have undergone acupuncture treatment within 1 month before participating. | | require |
| 11 | Pregnant and lactating women or women who have recently had childbirth need without contraception. | | require |
| 12 | Patients with contraindications for MRI scanning (prostheses with metal fillers, tight space syndrome, etc.). | | require |
| 13 | Patients with a weight change of more than 5% in the 8 weeks before randomization. | | require |
| 14 | According to the researcher's judgment, it is not suitable to participate in the research. | | require |

**Table S2. Locations of non-acupoints in sham acupuncture (SA) group**

| **Non-Acupoints** | **Sides** | **Location** |
| --- | --- | --- |
| NA1 | bilateral | On the same level of the 4 cun above the umbilicus, and 1 cun bilateral to the anterior midline. |
| NA2 | bilateral | On the same level of the umbilicus, and 1 cun lateral to the anterior midline. |
| NA3 | bilateral | On the same level of the 3 cun below the umbilicus, and 1 cun lateral to the anterior midline. |
| NA4 | bilateral | 2 cun above the anterior superior iliac spine |
| NA5 | bilateral | The ulnar margin of forearm, the midpoint of the line between the medial epicondyle of humerus and the styloid process of ulna |
| NA6 | bilateral | Lateral leg，3 cun below Yanglingquan (GB34)，between GB and BL |
| NA7 | bilateral | Lateral leg, 6 cun above the tip of the lateral ankle, 4 transverse fingers from the front edge of the tibia. |
| NA8 | bilateral | Midpoint of Sanyinjiao (SP6) Taixi (KI3) connection. |

**Table S3. Locations of acupoints in electro-acupuncture (EA) group**

| **Acupoints** | **Sides** | **Angle** | **Location** |
| --- | --- | --- | --- |
| CV12 Zhongwan | only middle | straight | On the anterior midline, 4 cun above the umbilicus. |
| CV4  Guanyuan | only middle | straight | On the anterior midline, 3 cun below the umbilicus. |
| ST25 Tianshu | bilateral | straight | On the same level of the umbilicus, and 2 cun bilateral to the anterior midline. |
| SP15  Daheng | bilateral | straight | On the same level of the umbilicus, and 4 cun bilateral to the anterior midline. |
| LV13  Zhangmen | bilateral | oblique | On the bilateral region of abdomen, under the tip of the elbow when the shoulder is dropped, and the elbow joint is flexed. |
| ST36  Zusanli | bilateral | straight | 3 cun directly below Dubi (ST35), and one finger-breadth bilateral to the anterior border of the tibia. |
| SP6  Sanyinjiao | bilateral | straight | On the medial aspect of the lower leg, 3 cun above the medial malleolus, on the posterior border of the medial aspect of the tibia. |
| LI4  Hegu | bilateral | straight | On the back of the hand, between the first and second metacarpals, at the midpoint of the radial side of the second metacarpal. |
| LV3 Taichong | bilateral | straight | On the dorsal side of the foot, the depression before the junction of the first and second metatarsals. |

**Table S4. Similarities and differences between two acupuncture groups**

|  | **Sham acupuncture**  **(SA)** | **Electro-acupuncture**  **(EA)** |
| --- | --- | --- |
| Kinds of points stimulated | Non-acupoints | Acupoints |
| Number of acupoints/non-acupoints | 16 | 16 |
| Depth of penetration, mm | 2-3 | ≥ 20 |
| De qi | No | Yes |
| Electro-acupuncture apparatus | attached | attached |
| Electric current | No | Yes |
| Needle retention, min | 30 | 30 |
| Number of sessions | 36 | 36 |

**Table S5. Procedure of enrollment, intervention and assessments**

|  | **STUDY PERIOD** | | | | | | |
| --- | --- | --- | --- | --- | --- | --- | --- |
|  | Baseline | Allocation | Intervention  (12 weeks) | | | | Follow-up  (4 weeks) |
| TIME POINT |  | W0 | AFT | W4 | W8 | W12 | W16 |
| **ENROLLMENT** | | | | | | | |
| Eligibility screening | X |  |  |  |  |  |  |
| Informed consent | X |  |  |  |  |  |  |
| Randomization | X |  |  |  |  |  |  |
| **INTERVENTIONS** | | | | | | | |
| SA group |  |  | | | | |  |
| EA group |  |  | | | | |  |
| **ASSESSMENTS** | | | | | | | |
| ***Primary outcome*** | | | | | | | |
| MRI-PDFF | X |  |  |  |  | X | X |
| ***Secondary outcomes*** | | | | | | | |
| MRE | X |  |  |  |  | X | X |
| Biochemical varieties | X |  |  |  |  | X |  |
| Anthropometry Parameters | X |  |  |  |  | X | X |
| ***Exploratory outcomes*** | | | | | | | |
| AIS-8, PHQ-2, GAD-2 | X |  |  |  |  | X |  |
| TFEQ-21, IPAQ-7, URICA | X |  | X | X | X | X | X |
| CLDQ-NAFLD/NASH | X |  | X | X | X | X | X |
| Blinding assessment |  |  | X |  |  | X |  |
| Credibility/expectancy  questionnaire |  |  | X |  |  | X |  |
| ***Safety outcome*** | | | | | | | |
| Adverse Events |  |  | X | X | X | X |  |

Abbreviations: AFT, After the First Treatment

**Table S6. Treatment primary outcome of patients with NASH in PP database**

|  | **Electro-acupuncture**  **n=26** | | **Sham acupuncture**  **n=25** | ***p* value** |
| --- | --- | --- | --- | --- |
| **Relative liver fat reduction from baseline in MRI-PDFF%, %** | | | | |
| Week 12 | | -33.2 (-47.7, -22.7) | -16.6 (-35.8, -22.7) | 0.021* |
| Week 16 | | -41.6 (-59.9, -19.3) | -14.4 (-59.9, -19.3) | 0.002* |
| **Patients with a ≥ 30% relative decline in MRI-PDFF%, n (%)** | | | | |
| Week 12 | | 16/26 (61.5%) | 7/25 (28.0%) | 0.016* |
| Week 16 | | 18/26 (69.2%) | 10/25 (40.0%) | 0.036* |

**Table S7. Change from baseline to week 12 in liver and metabolic biomarkers in PP database**

|  | **Electro-acupuncture**  **n=30** | **Sham acupuncture**  **n=27** | ***p* value** |
| --- | --- | --- | --- |
| **Non-invasive test** |  |  |  |
| Liver stiffness (MRE), kPa | -0.1 (-0.4, 0.3) | 0.1 (-0.2, 0.6) | 0.261 |
| **Biochemical varieties** |  |  |  |
| ALT, U/L | -53.0 (-71.5, -27.5) | -35.0 (-50.0, -22.0) | 0.119 |
| AST, U/L | -25.1 (-41.9, -15.2) | -17.8 (-24.7, -7.3) | 0.041* |
| GGT, mmol/L | -14.0 (-26.0, -5.5) | -13.0 (-24.0, -7.0) | 0.806 |
| ALP, mmol/L | -2.0 (-7.5, 5.5) | 0.0 (-11.0, 6.0) | 0.940 |
| CHO, mmol/L | 0.0 (-0.9, 0.2) | 0.0 (-0.6, 0.5) | 0.479 |
| TG, mmol/L | -0.4 (-0.8, 0.1) | -0.1 (-0.4, 0.3) | 0.151 |
| LDL-C, mmol/L | 0.0 (-0.4, 0.3) | -0.1 (-0.5, 0.3) | 0.692 |
| UA, µmol/L | -30.9 (-66.0, 50.9) | -39.4 (-75.3, 0.4) | 0.157 |
| FBG, mmol/L | -0.3 (-0.2, 1.5) | -0.2 (-0.4, 0.2) | 0.082 |
| HbA1c% | -0.2 (-0.3, -0.1) | 0.0 (-0.3, 0.1) | 0.064 |
| HOMA-IR | -2.5 (-5.7, -0.7) | -1.3 (-3.5, 0.3) | 0.083 |

**Table S8. Blinding assessment by patients assessed**

|  |  | **Sham acupuncture**  **(SA)** | | **Electro-acupuncture**  **(EA)** | | ***p* value** |
| --- | --- | --- | --- | --- | --- | --- |
| **Blind Test**  **Accordance，**  **n（%）** | After 1^th^ | | 18/27 (66.7%) | | 11/30 (36.7%) | 0.697 |
|  | Week 12 | | 20/25 (80.0%) | | 8/26 (30.8%) | 0.000* |

**Table S9. Credibility/expectancy questionnaire in patients**

|  |  | **Sham acupuncture**  **SA** | **Electro-acupuncture**  **EA** | ***p* value** |
| --- | --- | --- | --- | --- |
| **CEQ score** | After 1^th^ | 8.8 $\pm$ 0.9  (n=27) | 8.9 $\pm$ 1.2  (n=30) | 0.725 |
|  | Week 12 | 8.9 $\pm$ 2.0  (n=25) | 9.4 $\pm$ 0.9  (n=26) | 0.174 |
