# Supplementary figures and images for: Electro-acupuncture reduced steatosis on MRI-PDFF in patients with non-alcoholic steatohepatitis: a randomized controlled pilot clinical trial
Source: Chin Med. 2023 Feb 24;18:19. doi: 10.1186/s13020-023-00724-w (PMC9950708; doi:10.1186/s13020-023-00724-w)

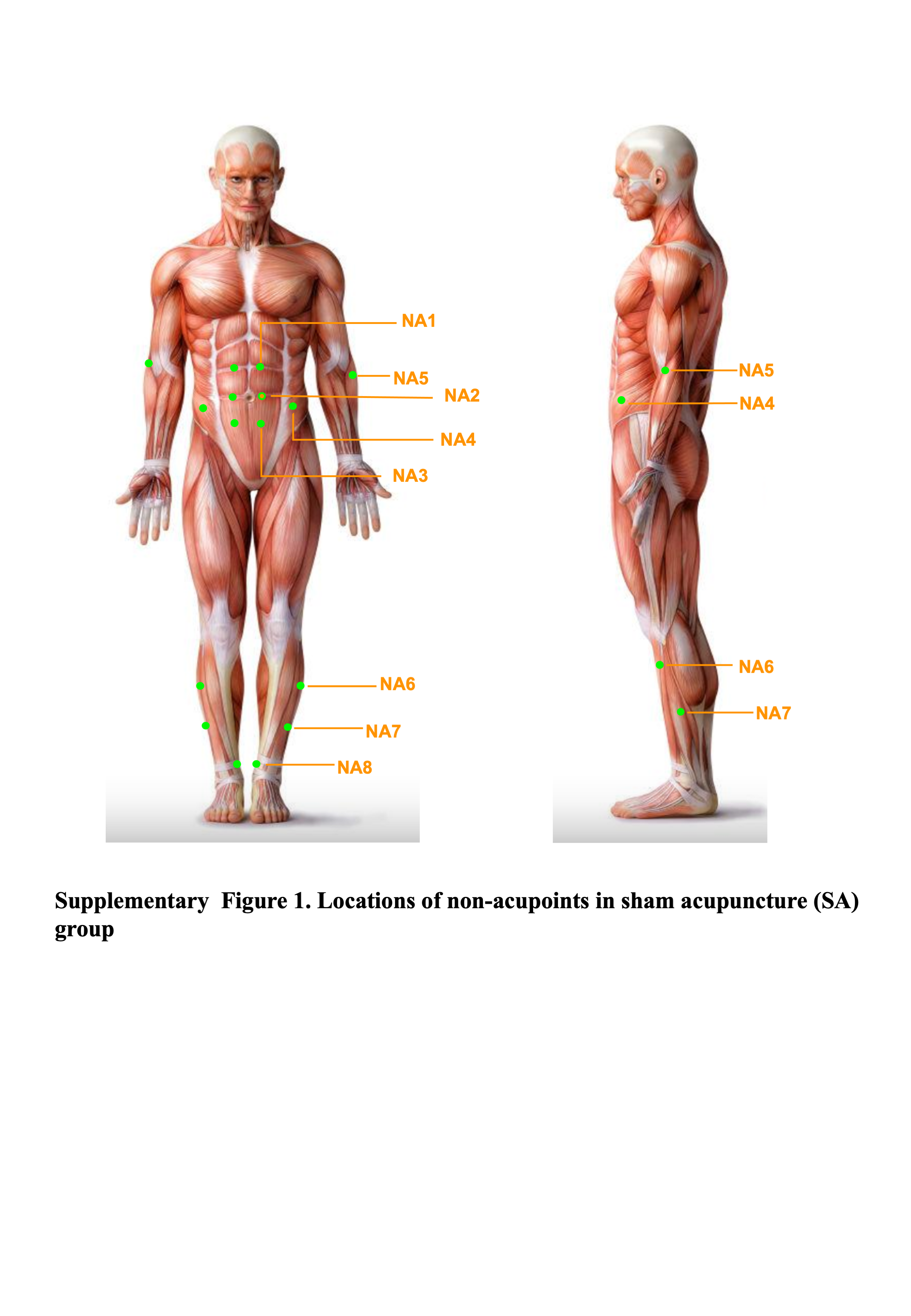

Supplement: Supplementary file 2 — Additional file 2: Locations of non-acupoints in sham acupuncture(SA) group. [file 13020_2023_724_MOESM2_ESM.tiff]

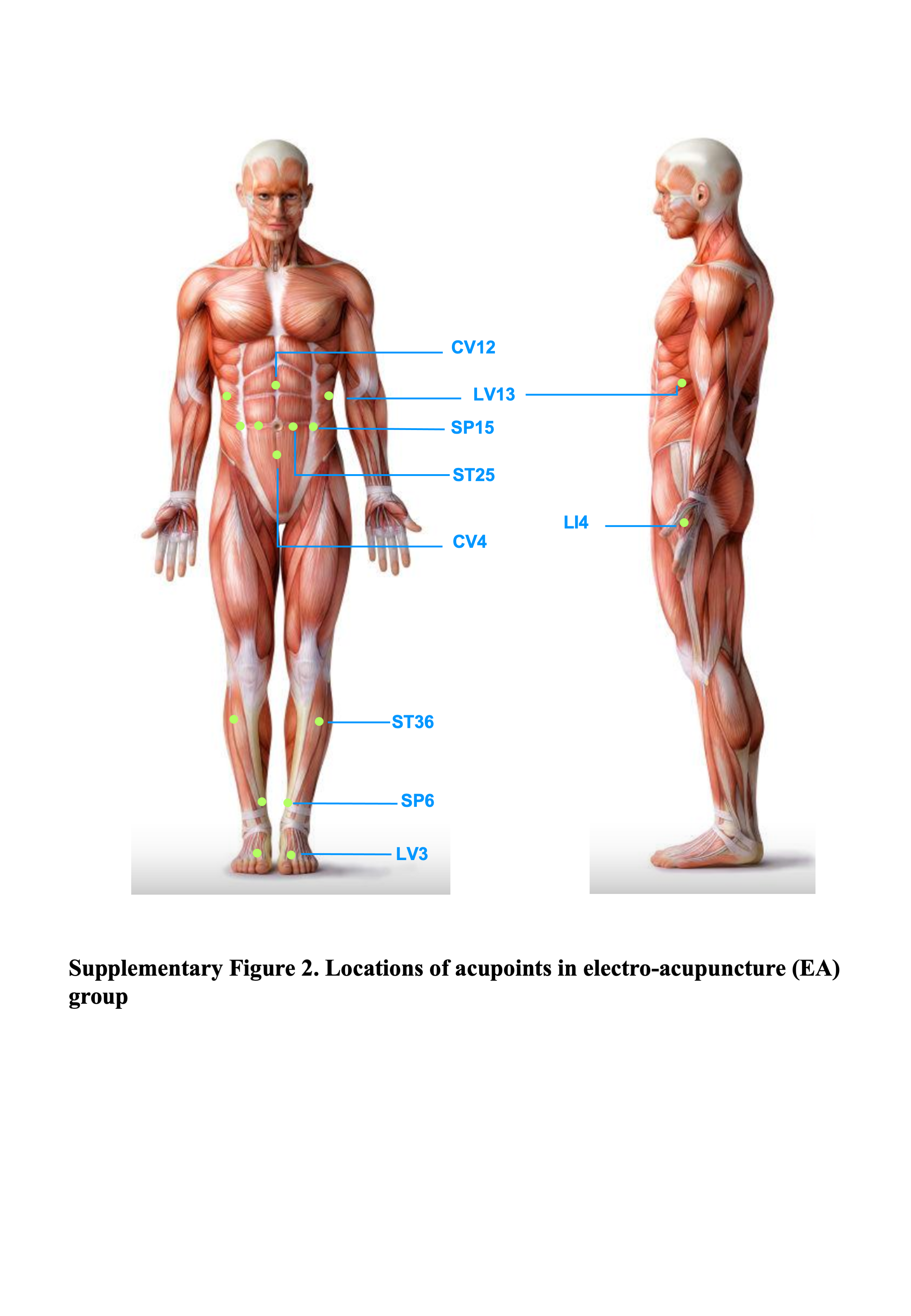

Supplement: Supplementary file 3 — Additional file 3: Locations of acupoints in electro-acupuncture(EA) group. [file 13020_2023_724_MOESM3_ESM.tiff]

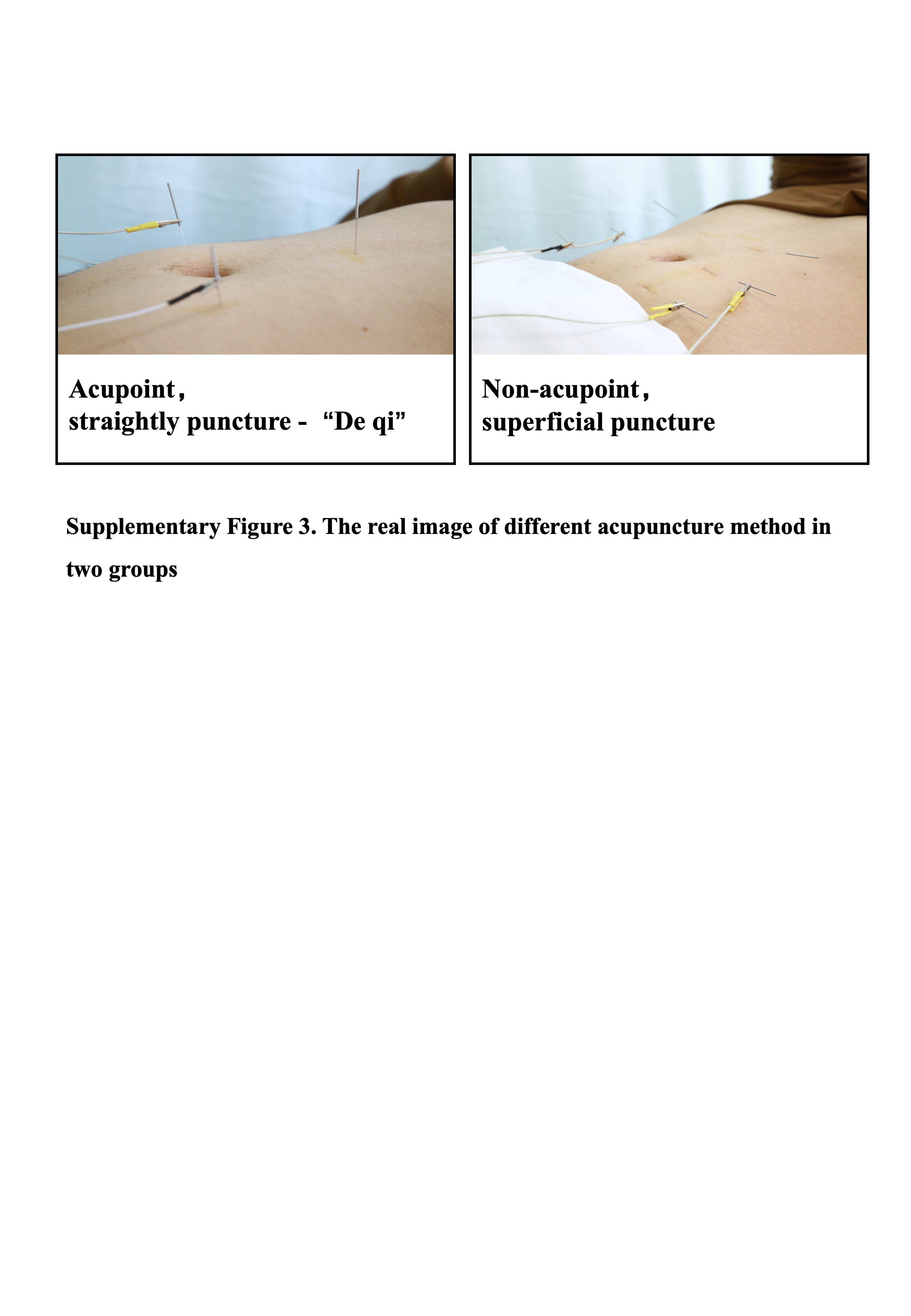

Supplement: Supplementary file 4 — Additional file 4: The real image of different acupuncture method in two groups. [file 13020_2023_724_MOESM4_ESM.tiff]

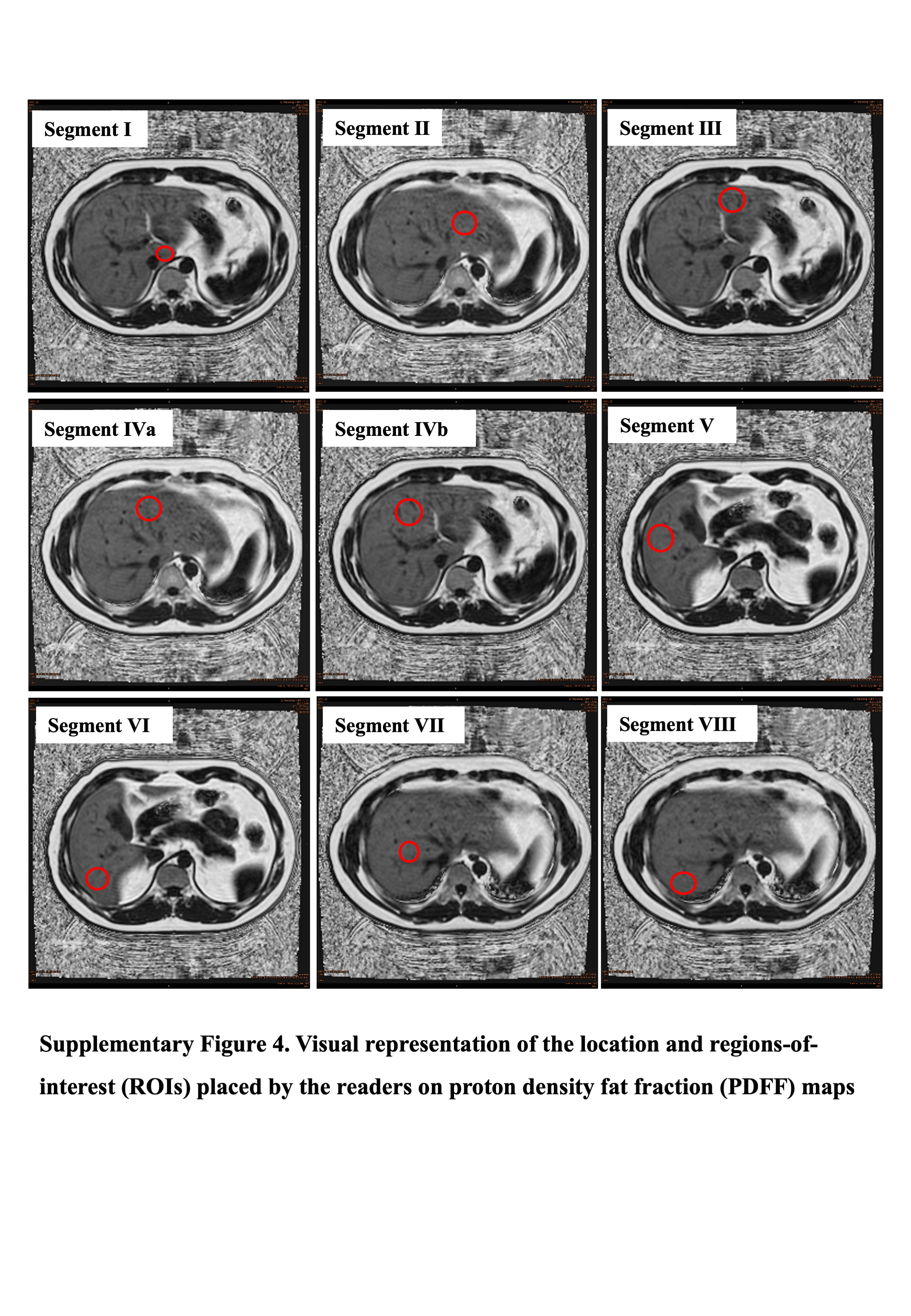

Supplement: Supplementary file 5 — Additional file 5: Quality control of MRI-PDFF. [file 13020_2023_724_MOESM5_ESM.tiff]

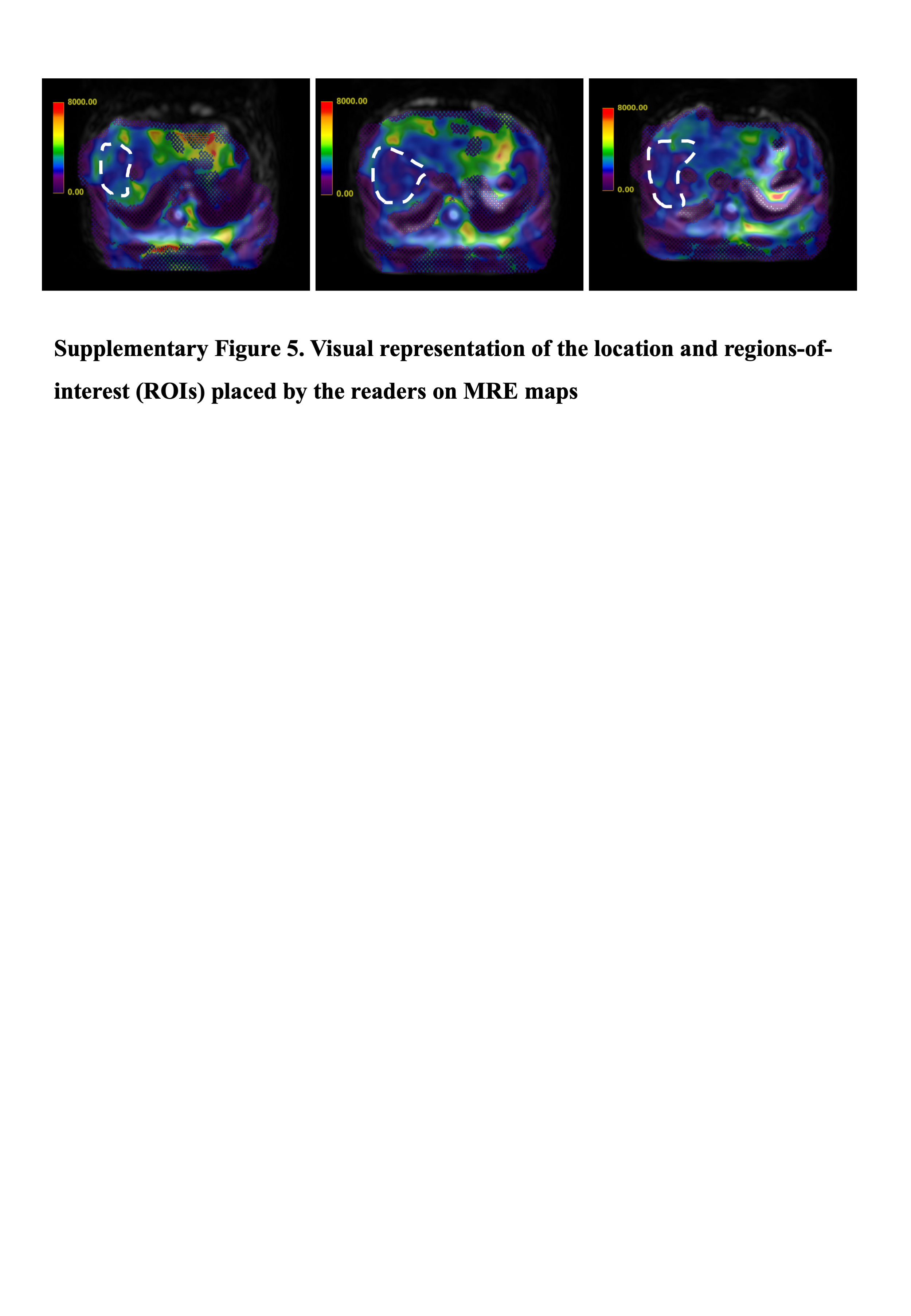

Supplement: Supplementary file 6 — Additional file 6: Quality control of MRE. [file 13020_2023_724_MOESM6_ESM.tiff]
